# Honey bees (*Apis cerana*) use animal feces as a tool to defend colonies against group attack by giant hornets (*Vespa soror*)

**DOI:** 10.1371/journal.pone.0242668

**Published:** 2020-12-09

**Authors:** Heather R. Mattila, Gard W. Otis, Lien T. P. Nguyen, Hanh D. Pham, Olivia M. Knight, Ngoc T. Phan

**Affiliations:** 1 Department of Biological Sciences, Wellesley College, Wellesley, Massachusetts, United States of America; 2 School of Environmental Sciences, University of Guelph, Guelph, Ontario, Canada; 3 Insect Ecology Department, Institute of Ecology and Biological Resources, Vietnam Academy of Science and Technology, Hanoi, Vietnam; 4 Bee Research Centre, National Institute of Animal Sciences, Hanoi, Vietnam; 5 Research Center for Tropical Bees and Beekeeping, Vietnam National University of Agriculture, Hanoi, Vietnam; Universitat Leipzig, GERMANY

## Abstract

Honey bees (genus *Apis)* are well known for the impressive suite of nest defenses they have evolved to protect their abundant stockpiles of food and the large colonies they sustain. In Asia, honey bees have evolved under tremendous predatory pressure from social wasps in the genus *Vespa*, the most formidable of which are the giant hornets that attack colonies in groups, kill adult defenders, and prey on brood. We document for the first time an extraordinary collective defense used by *Apis cerana* against the giant hornet *Vespa soror*. In response to attack by *V*. *soror*, *A*. *cerana* workers foraged for and applied spots of animal feces around their nest entrances. Fecal spotting increased after colonies were exposed either to naturally occurring attacks or to chemicals that scout hornets use to target colonies for mass attack. Spotting continued for days after attacks ceased and occurred in response to *V*. *soror*, which frequently landed at and chewed on entrances to breach nests, but not *Vespa velutina*, a smaller hornet that rarely landed at entrances. Moderate to heavy fecal spotting suppressed attempts by *V*. *soror* to penetrate nests by lowering the incidence of multiple-hornet attacks and substantially reducing the likelihood of them approaching and chewing on entrances. We argue that *A*. *cerana* forages for animal feces because it has properties that repel this deadly predator from nest entrances, providing the first report of tool use by honey bees and the first evidence that they forage for solids that are not derived from plants. Our study describes a remarkable weapon in the already sophisticated portfolio of defenses that honey bees have evolved in response to the predatory threats they face. It also highlights the strong selective pressure honey bees will encounter if giant hornets, recently detected in western North America, become established.

## Introduction

Social insects house families in centralized, resource-rich nests, a trait that has driven the evolution of defense strategies to counteract attempts by thieves and predators to exploit their bounty. Nest defense can take the form of physical, chemical, and behavioral barriers, the combination of which are known as defense portfolios [1, 2]. Honey bees (genus *Apis*), famous for the resources that their prolific colonies sequester, provide an impressive example of the diversity of nest-defense strategies that have evolved within a geographically widespread group [3, 4]. Because of their ability to respond to threats collectively, honey bees exhibit varied and often spectacular suites of defensive responses to counteract the predatory pressures that different species face [3–5]. Honey bee nests attract an array of vertebrate and invertebrate predators, most of which are larger than bees themselves and attack colonies to prey upon adults and brood and steal stored food [4]. For honey bees, eusocial wasps in the genus *Vespa* (the true hornets) are a particularly dangerous category of predator that exert tremendous selective pressure on their prey [5–10]. Hornets hunt for insects to feed offspring that demand a continuous source of fresh prey, which can be provided in abundance by the resources in a honey bee nest [8, 11–13].

*Vespa* hornets are superbly adapted for hunting insects like bees: they are typically larger than their victims, they have strong mandibles for crushing, dismembering and masticating prey, and, while armed with a venomous sting, they are well-armored to avoid stings themselves [7, 11, 14]. While there is evidence that honey bees may try to use their sting against hornets [10, 15], they are often ineffective when attackers are large [16–18]. Hornet attacks on nests range from solitary individuals hunting bees near the ground, darting in from perches near entrances, and hawking bees from the air [9, 10, 14, 19] to mass slaughter of bees and occupation of their nests by groups of hornets [7, 8, 20]. Persistence using any of these approaches can result in debilitation or death of a colony [7, 8, 10].

In the arms race to counteract these potentially devastating attacks by hornets, many coordinated group defenses have evolved across different species of *Apis* [5]. Some strategies are similar across species, while others are unique to specific taxa. The first line of defense is physically shielding the colony from predators. Cavity-nesting species find protection behind enclosed walls and a small entrance that is monitored by guards, while more exposed open-nesting species rely on a curtain of defensive workers that envelopes their comb [5, 21]. Synchronized body shaking or wave-like visual displays that warn and repel approaching predators are widespread among *Apis* species [14, 22–28]. Many species of honey bees produce hissing or buzzing sounds in response to predators [5, 29–31], aposematic signals that can serve as deterrents to nest intruders [32]. Killing hornets by overheating and suffocating individual attackers in a ball of bees is a defense that is employed pervasively across honey bee species [5, 10, 14, 17–20, 26, 33, 34]. These coordinated defensive behaviors are most strongly expressed by honey bees that have evolved under a high degree of predatory pressure from hornets, the distributions of which are restricted mostly to Asia [35, 36]. For example, colonies of *A*. *cerana* are beleaguered by several hornet species that are endemic throughout this species’ vast range in Asia [5, 22, 37, 38] and, in response, *A*. *cerana* workers form hotter and larger balls and kill more hornets under natural conditions than *A*. *mellifera* workers, which evolved outside of Asia and under much lower levels of hornet predation [34, 39]. As a result, hornets preferentially prey on colonies of *A*. *mellifera* wherever they have been introduced into *A*. *cerana*’s range [6, 8, 9]. However, some subspecies of *A*. *mellifera* (e.g., *A*. *m*. *cypria*) ball hornets more effectively than others, likely because this subspecies coevolved with a sympatric hornet, *Vespa orientalis* [17, 26]. The destructive potential of hornet attacks and the corresponding importance of co-evolved defense strategies is revealed by a striking inability of honey bees to fend off attacks when either predator or prey is introduced into the other’s range [8, 9, 12, 13, 39, 40].

One of the best characterized and most dramatic of these bee-hornet interactions involves honey bees and *Vespa mandarinia*, whose range overlaps broadly in temperate regions of Asia with both introduced and native honey bee species [8, 35, 38, 41, 42]. Workers of *V*. *mandarinia* hunt individual honey bees, but they are most deadly when they target an entire colony for takeover [7, 8, 11]. In a successful attack, a scout hornet first chemically marks the colony’s nest and then she recruits, through unknown means, up to 50 nestmates to the site [7]. The attacking hornets, which are many times larger than the bees, grab defending workers and kill them one after another. During this slaughter phase, each hornet can kill thousands of bees and, collectively, a group of hornets can obliterate a colony’s defensive force within a few hours [7, 8]. When resistance ends, the occupation phase begins: hornets enter the nest, begin guarding it as their own, and shuttle brood back to their own nest to feed their young [7]. *V*. *mandarinia* can easily overtake colonies of *A*. *mellifera* and smaller social wasp species [8, 11], but their attacks face greater resistance from *A*. *cerana* colonies [7, 11]. *A*. *cerana* workers often thwart potential extermination of their colony using a suite of known defensive behaviors. When potential hornet scouts are detected, *A*. *cerana* workers retreat into nests, produce vibratory signals that encode the severity of threat at the nest entrance, and stimulate their nestmates (in part by the release of alarm pheromone) to prepare to “heat ball” scouting hornets [7, 11, 20, 43, 44]. In Japan, *A*. *c*. *japonica* has been documented waggle dancing at hive entrances after exposure to tethered *V*. *mandarinia*, a behavior that stimulates foragers to collect and smear plant-based materials around nest entrances, possibly interfering with pheromones deposited by hornet scouts [45, 46].

We explore for the first time the defensive response of *Apis cerana* (Fabricius, 1793) to a similarly fearsome, but poorly studied, hornet predator: *Vespa soror* (du Buysson, 1905). *V*. *soror*, the closely related sister species of *V*. *mandarinia*, occurs in southern China and subtropical regions of Southeast Asia [35, 36, 41, 42, 47–49]. Once considered a subspecies of *V*. *mandarinia*, *V*. *soror* has been elevated to a separate species, in part because it is sympatric with *V*. *mandarinia* where the ranges of the two species overlap [35, 41]. We conducted our studies in Vietnam, where *V*. *soror* is common and *V*. *mandarinia* is rare [42, 48]. Little is known about the biology of *V*. *soror*, but it has been observed in Hong Kong to have predatory habits that are similar to *V*. *mandarinia*: it launches group attacks on nests of other social wasps and has been reported by beekeepers to slaughter and then occupy managed honey bee colonies [50]. Nest structure reveals that the species share similar nesting habits and their colonies are equivalently large at maturity (typically 2,700–3,700 cells, with ~600 morphologically similar workers and reproductives [7, 41, 50]).

In Vietnam, we heard repeatedly from beekeepers of attempts by *V*. *soror* to attack *A*. *cerana* colonies. Prior to this study, we observed spots of unknown material around hive entrances during visits to apiaries that beekeepers consistently stated was the bees’ response to attack by hornets. One beekeeper said that the spots were animal feces because he had observed bees collecting water buffalo dung. Presently, all honey bees are considered to forage exclusively for materials produced by plants (e.g., nectar, pollen, resin) and water-associated fluids (with the exception of beeswax salvaging by *Apis florea* [51]). These fluids can be from dirty sources, including brackish water, sweat, runoff from animal feces, and vertebrate urine, which are presumed to contain attractive nutrients [52–55]. Bumblebees (*Bombus*) and stingless bees (Meliponini) also drink fluids from filth [56, 57], the term used to describe excrement, carrion, or other decaying material when it is used as a resource by insects [56, 58]. Although several species of stingless bees collect vertebrate feces and incorporate it into nests [56, 59, 60], collection of solid feces is unknown in honey bees (*Apis*).

We investigated the potential use of feces by *A*. *cerana* to defend their nests against attack by *V*. *soror*, which we confirmed preys upon honey bee colonies *en masse*, like *V*. *mandarinia*. Through a series of field experiments, we show that workers of *A*. *cerana* respond to this lethal threat by foraging for animal feces (and other filth) and applying it around nest entrances. We show that entrance spotting by *A*. *cerana* is geographically widespread, that it is a response to attack by *V*. *soror* workers specifically (and not to predation by another less deadly *Vespa* predator), that nest-marking chemicals (in the absence of hornets) elicit the collection and application of fecal spots to the bees’ nests, and that the bees’ behaviors decrease the severity of attack by repelling hornets from nest entrances, where attacks were focused. We report for the first time the remarkable employment by *A*. *cerana* of feces to defend their nests, a behavior that constitutes the first report of honey bees using a tool–a non-plant solid–to deter attack by a dangerous predator.

## Results

### Fecal spotting by *Apis cerana* is widespread

**Fig 1:**
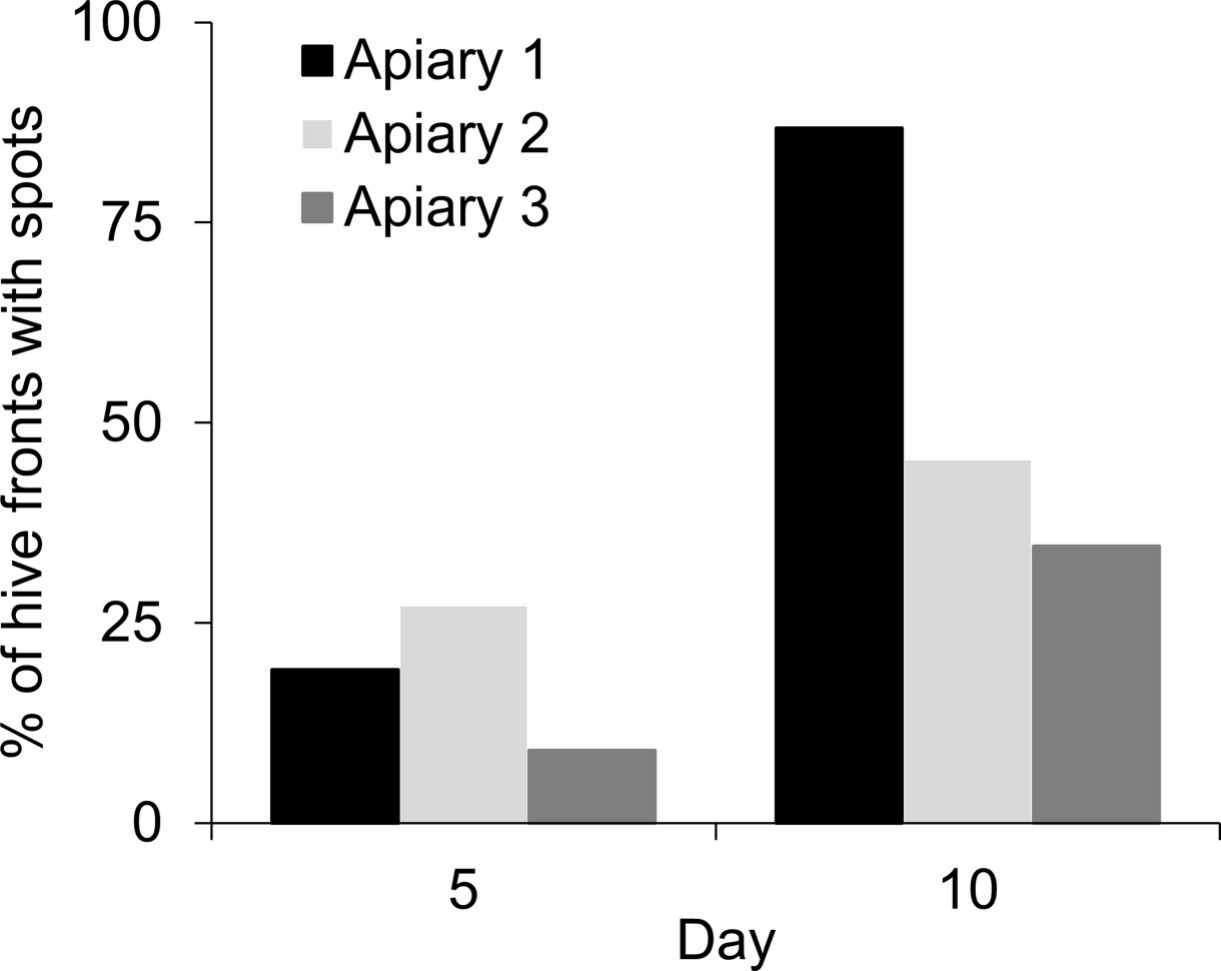
Spotting on hive fronts increased over time. The hive fronts of a total of 339 colonies in 3 apiaries were washed with water to remove spots and the presence or absence of spots on hive fronts was evaluated 5 and 10 days later.

By monitoring colonies over 10 days, we determined that a high percentage of colonies had accumulated fresh spots of material on their hive fronts, this material was concentrated around entrances, and that workers applied the spots. The latter was clear from day-long videos that repeatedly showed workers applying spots, each individual standing with her antennae directed toward her hive’s surface and rocking her head against the hive. The percentage of colonies with spots on their hive fronts increased over the 10-day observation period, although the occurrence of spotting differed among the three apiaries (Fig 1; 2x3 contingency table; day 5: χ^2^ = 8.3, df = 2, p = 0.02; day 10: χ^2^ = 68.9, df = 2, p < 0.0001). The spots were applied around hive entrances, with their spread encompassing entrance openings in almost all cases (99.0%). In general, spots were not applied far from entrances; the furthest spots were 6.4 ± 0.3 cm from the nearest margin of the entrance and were applied within an area of 105 ± 8 cm^2^ (means ± SEMs).

**Fig 2:**
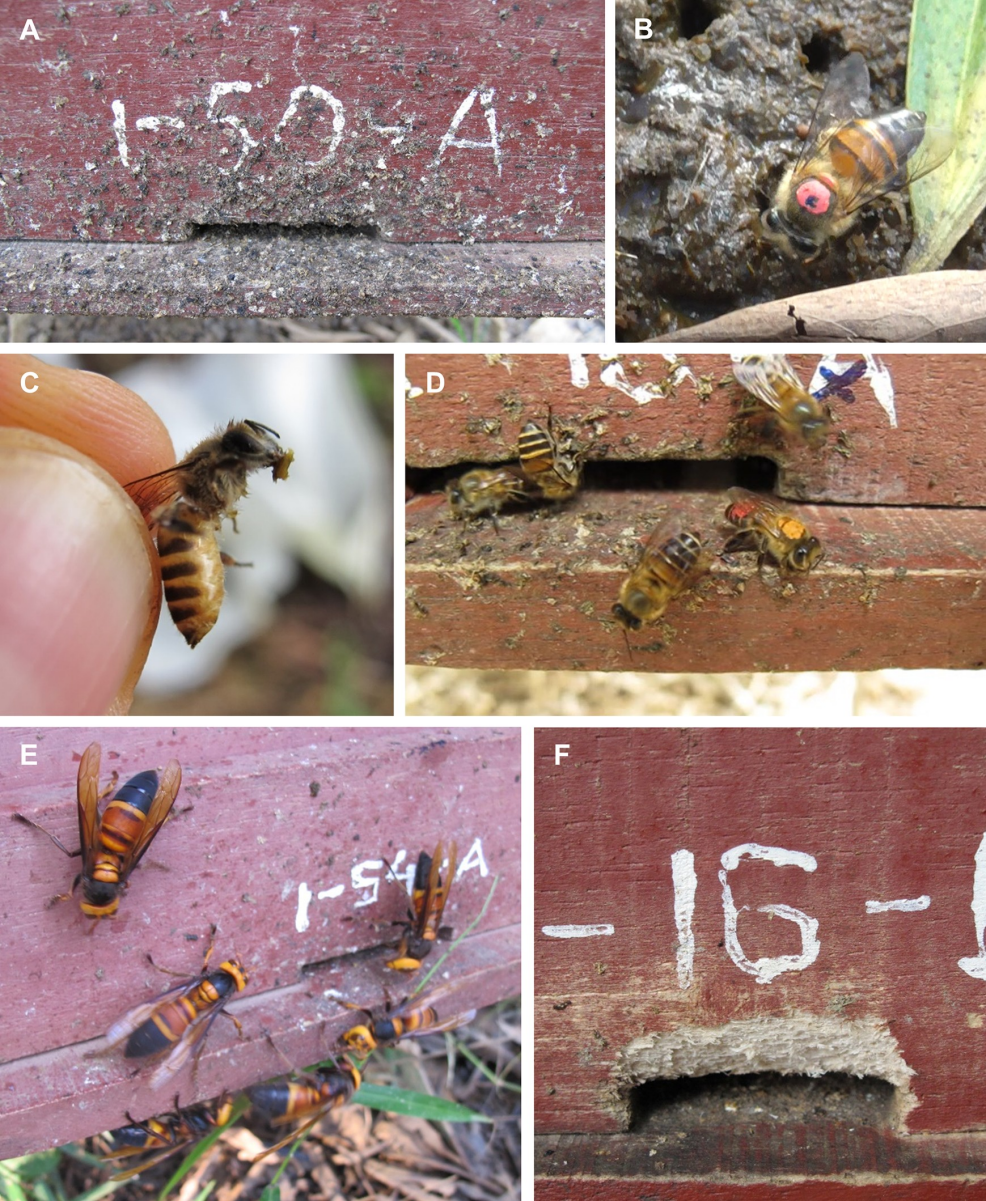
*Apis cerana* defended their colonies from group attack by *Vespa soror* by applying fecal spots around hive entrances. (A) A hive front with heavy fecal spotting around the entrance opening. (B) A marked *A*. *cerana* forager on a dung pile. (C) A forager holding a clump of fecal solids in her mandibles, captured after leaving a dung pile. (D) A forager applying a fecal spot to a hive front after being paint marked on a dung pile. (E) An entrance-focused group attack on a colony by six *V*. *soror* workers. (F) Damage to a hive entrance after entrance margins were chewed on by *V*. *soror* workers (the attack was stopped by experimenters before the nest was breached).

Field observations revealed that *A*. *cerana* workers sought out animal feces, carried it back to their colonies, and then applied it as textured spots on the fronts of hives (Fig 2A–2D). Foragers landed on and flew between the dung piles that we placed beside Apiary 1. Once landed, a forager focused on a particular site on a pile, pulled at fecal material and worked it with her mandibles, and eventually flew away with a small clump of feces in her mouthparts (S1 and S2 Videos). We never saw workers extend their proboscises to ingest fluids from dung. Marked foragers were observed returning to their hives with fecal clumps and applying them as mounded (not smeared) spots to hive fronts and landing boards. Fecal spots were predominantly various shades of brown and gray and had three-dimensional texture. Foragers that applied fecal spots stood with their mouthparts and antennae oriented toward their hives’ surfaces and used their mandibles to shape clumps (S3 and S4 Videos). We occasionally saw foragers enter colonies with feces in their mandibles. We observed many marked foragers returning over multiple trips and days to locations on dung piles that they had visited before. Workers collected feces at our dung piles throughout the study; we also observed them foraging for feces in a nearby chicken coop. In addition to animal feces, we saw foragers collecting soap scum and one hive had spots applied to it that were bright blue, but we could not determine their origin. On one occasion, a hive smelled strongly of urine and we found workers visiting a container of human urine nearby.

We surveyed 72 Vietnamese beekeepers in late August, when attacks by *V*. *soror* are frequent (Fig 2E and 2F), to determine how widespread spotting was in Vietnam. Five beekeepers kept *A*. *mellifera* colonies only; these beekeepers did not observe spots on their hives (median 60, range 40–700 colonies per beekeeper). Of the 67 remaining beekeepers who kept *A*. *cerana* colonies, 63 of them (94%) reported spots on the fronts of their hives (S1 Fig). *A*. *cerana* beekeepers had a median of 15 colonies per beekeeper (range 3–170 colonies) and reported spots on an average of 74% of their hive fronts (range 10–100% of hives spotted per beekeeper). These beekeepers also reported high rates of absconding in response to hornet attack (26% of colonies in 2012 and 11% of colonies by the end of August in 2013). We confirmed that *A*. *cerana* colonies abscond in response to sustained attack by *V*. *soror*. One morning, we widened the entrance of a colony that had been attacked the previous day. Within 30 minutes, the colony was visited by a single *V*. *soror* scout and, 75 minutes later, the colony absconded while under attack by at least eight hornets, including two hornets that were balled by the bees. The hornets subsequently occupied the hive and, over 24 hours of monitoring, carried away bee brood during the day and remained in the hive overnight.

### *Apis cerana* responds with fecal spotting to attack by *Vespa soror*

#### Test 1: Fecal spotting during attack

**Fig 3:**
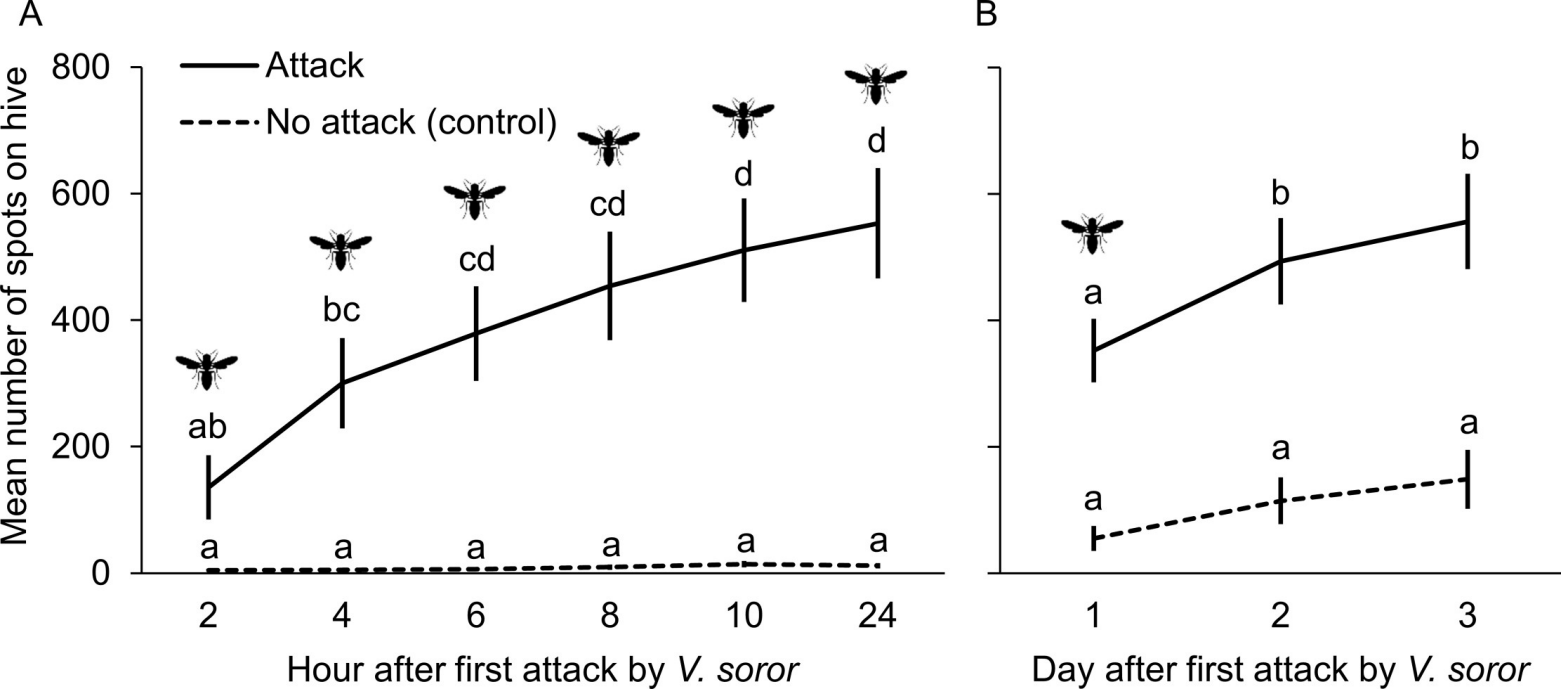
Fecal spotting on hive fronts increased in response to attacks by *Vespa soror*. In two tests, visits by hornets to attacked colonies occurred naturally over the course of a single day; hornets were prevented from approaching control colonies at all times. Hornet symbols signify treatment and time combinations during which hornets were permitted to attack. (A) Test 1: hive fronts were photographed at two-hour intervals during the day of attack and at the start of the next day to assess changes in spot number. (B) Test 2: hive fronts were photographed at the end of a day of attack and at the end of two subsequent days during which hornet attacks were prevented. Spot counts on hive fronts were estimated from photographs. All colonies were in the same apiary, but different colonies were used for each test. Different letters indicated significant differences in mean (± SEM) spot number across conditions.

We determined whether *A*. *cerana* colonies that were attacked by *V*. *soror* responded by spotting their hives. One set of colonies was visited throughout the day by *V*. *soror* hornets, while hornets were prevented by experimenters from approaching another set of control colonies. Colonies that experienced attacks had 12 ± 5 hornet visits per colony and total visit duration of 30 ± 18 minutes per colony (means ± SEMs). Mean spot number on the hive fronts of attacked colonies increased consistently for 24 h during these persistent attacks, whereas mean spot number on control colonies was low and failed to increase over the same period (Fig 3A; repeated measures ANOVA; treatment effect: F_1, 11_ = 291.5, p < 0.0001; time effect: F_5, 65_ = 8.8, p < 0.0001; interaction: F_5, 55_ = 8.0, p < 0.0001).

#### Test 2: Fecal spotting after attack

We determined with a different set of colonies whether a colony’s spotting response persisted even after hornet attacks ceased. When another day of hornet attacks was permitted for a second set of colonies, the number of spots on the fronts of their hives increased over the subsequent two days, even though hornet were prevented from attacking them over that period (Fig 3B; repeated-measures ANOVA; treatment effect: F_1, 26_ = 9.3, p = 0.005; time effect: F_2, 52_ = 12.2, p < 0.0001; interaction: F_2, 52_ = 1.7, p = 0.19). In comparison, the number of spots on the hive fronts of no-attack control colonies stayed consistently low throughout the duration of the test (Fig 3B). On average, the extent of hornet attacks in Test 1 did not differ from the extent of attacks in Test 2 (mean ± SEM: 21 ± 6 hornet visits per colony and total visit duration of 39 ± 11 minutes per colony; visits: t_32_ = 1.1, p = 0.27; duration: t_32_ = 0.4, p = 0.67).

#### Test 3: Fecal spotting in response to hornet species

We compared the spotting response of colonies to visits by workers of *V*. *soror* versus *Vespa velutina* (Lepeletier, 1836), a smaller hornet that hunts solitarily and while in flight [9]. On average, colonies visited by *V*. *soror* workers over two days had substantially higher numbers of spots on their hive fronts than colonies visited over the same period by *V*. *velutina* workers (mean ± SEM: 444 ± 73 versus 6 ± 3 spots per colony, respectively; t_39_ = 5.6, p < 0.0001). Our comparison of the attack behavior of these hornet species revealed that *V*. *soror* frequently landed on hives, and over half the time at hive entrances specifically (Fig 2E), whereas *V*. *velutina* landed on hives infrequently and hovered more than *V*. *soror* (Table 1; n = 857 and 328 visits, respectively). *V*. *soror* often chewed on the margins of entrance openings (Fig 2F) and rubbed their abdomen on hives, whereas *V*. *velutina* was never observed doing these behaviors (Table 1). Both species were equally as likely to interact with bees when visiting a colony, but visits by *V*. *soror* were far deadlier, resulting in bee death almost four times more often than visits by *V*. *velutina* (Table 1).

**Table 1:** *Vespa soror* attackers were more likely to land at and chew on entrances of hives than *Vespa velutina* attackers. Visits to *Apis cerana* colonies by *V*. *soror* (n = 857 visits) and *V*. *velutina* (n = 328 visits) hornets were observed in the same apiary and over six days per species. Attacks were monitored by observers stationed throughout the apiary and data were recorded only if hornet visits lasted at least 30 seconds and were observed from initial approach until departure from a hive. Asterisks indicate behaviors that are required to breach hive entrances and occupy nests.

Behavior during attack	% of total visits		|*z*| score	p-value
	*V*. *soror*	*V*. *velutina*		
Hovered in front of hive	65.3	93.3	9.7	< 0.0001
Landed anywhere on hive	64.4	7.0	17.7	< 0.0001
* Landed at entrance specifically	33.1	4.3	10.3	< 0.0001
* Chewed on margins of entrance	13.7	0	7.1	< 0.0001
Rubbed abdomen on hive	9.2	0	5.7	< 0.0001
Groomed self while landed	7.1	0	5.0	< 0.0001
Fanned wings while landed	11.0	0	6.3	< 0.0001
Trophallaxis with conspecific	3.0	0	3.2	0.001
Interacted with bee (chase or kill)	28.7	25.0	1.3	0.20
Killed bee	19.5	5.2	6.1	< 0.0001

#### Test 4: Fecal spotting in response to gland extracts

**Fig 4:**
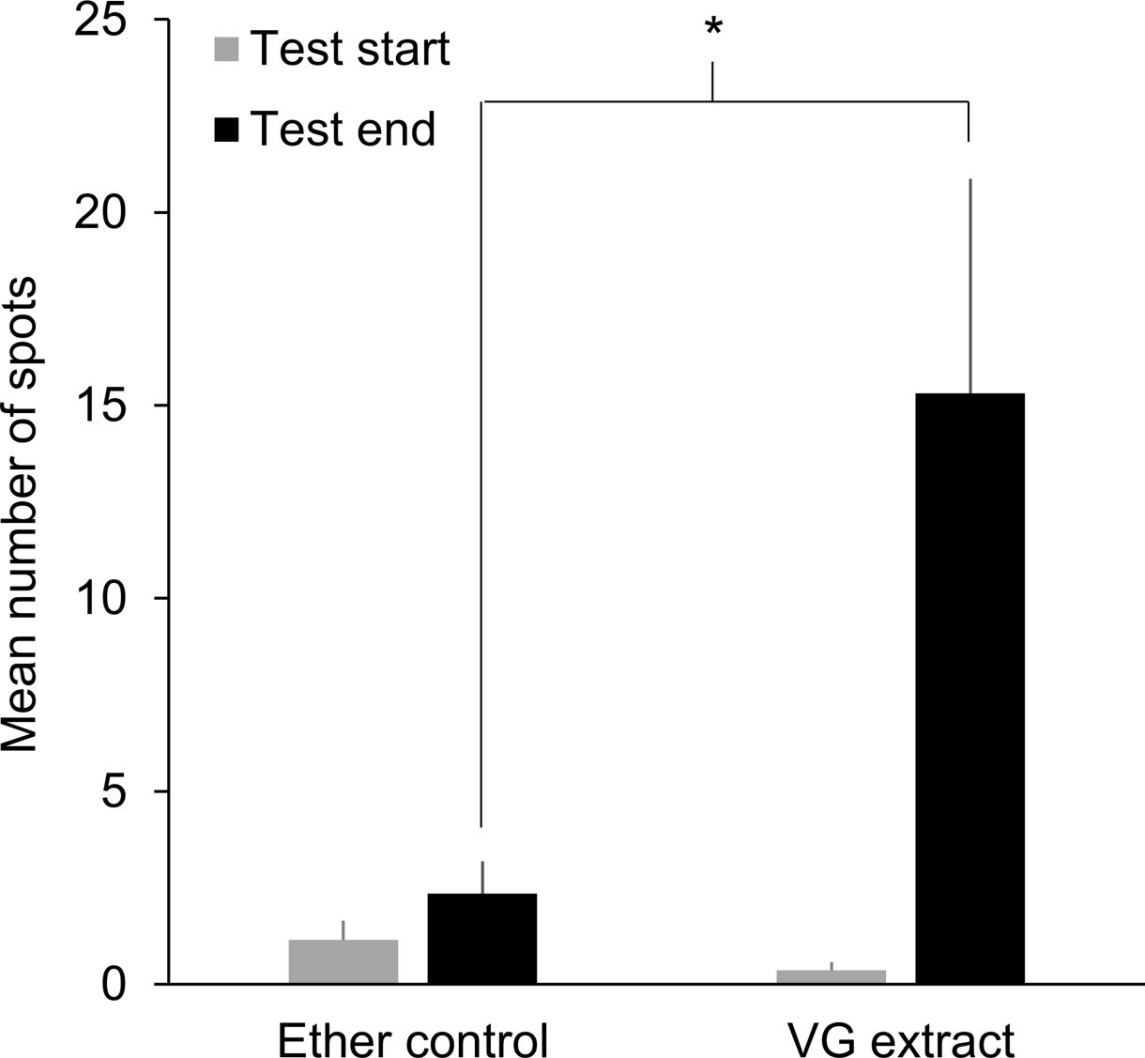
Fecal spotting on hive fronts increased after colonies were exposed to VG extracts. Each extract mixture was created by placing VGs from three *Vespa soror* workers into a vial with 0.5 mL ether for 24 hours (one vial per test colony). A 1 cm^2^ piece of filter paper was repeatedly soaked and dried in this mixture before it was pinned at a colony entrance; ether shams (filter papers without extracts) were created similarly and presented to colonies as a control. Hive fronts were photographed after six hours to determine changes in spot number. Asterisk indicates a significant difference between treatment means (± SEM) by the end of the test (p = 0.02).

**Fig 5:**
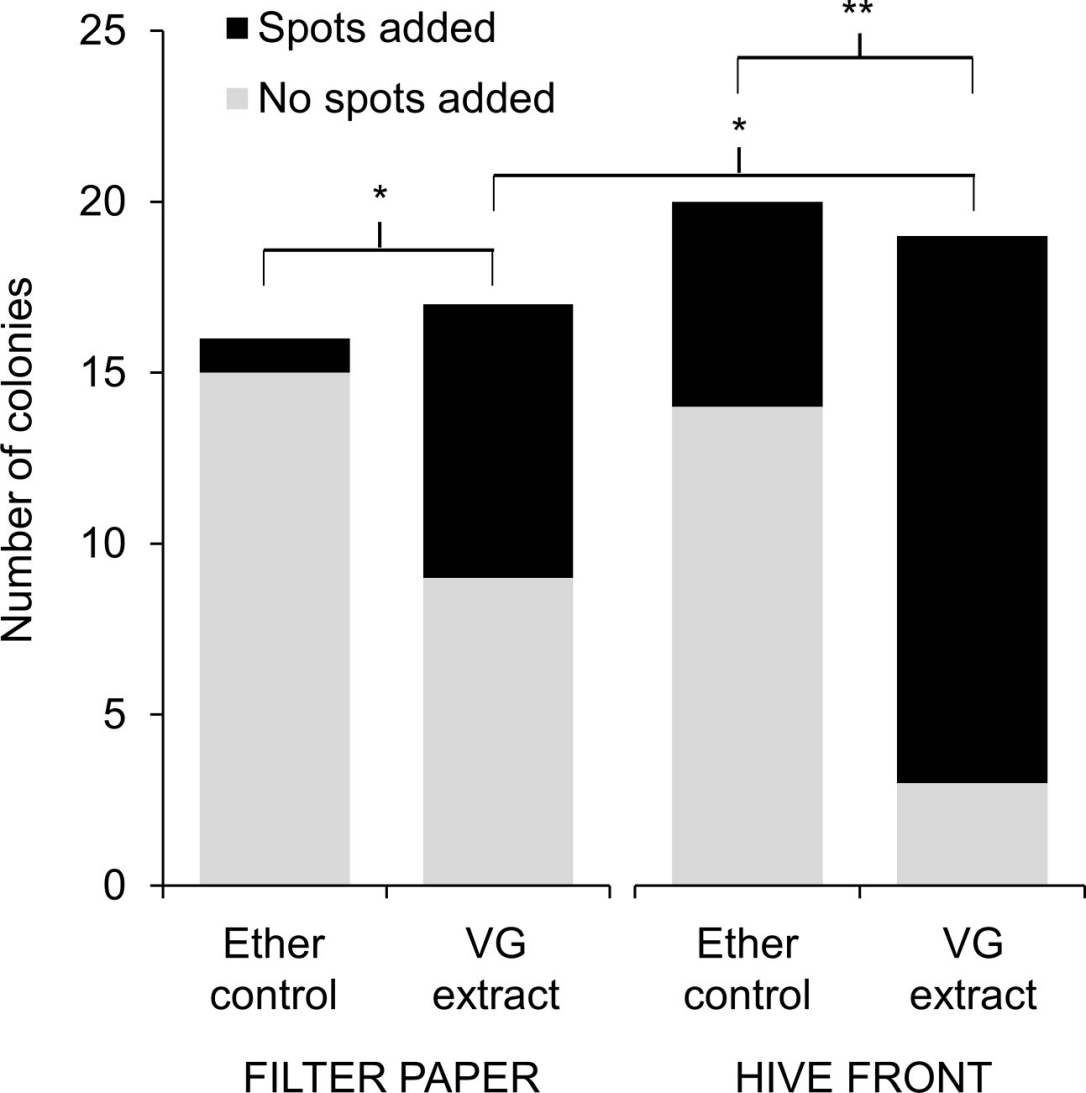
Exposure to VG extracts from *Vespa soror* workers induced spotting of hive fronts by most colonies. The addition of new spots to filter papers and hive fronts was determined six hours after colonies were exposed to filter paper soaked either in VG extract or in ether only (a sham control); refer to Fig 4 for details about the creation and presentation of VG extract-soaked and control filter papers. Asterisks indicate a significant difference between comparison groups, which are identified by brackets (* p ≤ 0.03; ** p = 0.001).

Lastly, we determined whether *A*. *cerana* colonies initiated spotting when presented with extracts from van der Vecht glands (VG), the putative source of pheromones that hornet workers use to mark target colonies prior to a mass attack [20]. Hives had similarly low numbers of spots at the start of the test, but after 6 hours of exposure to filter papers that were soaked in VG extract and then placed at their entrances, workers applied significantly more spots to their hive fronts compared to colonies exposed to ether shams as a control (Fig 4; t-test; start: t_37_ = 1.4, p = 0.16; end: t_37_ = 2.4, p = 0.02). A greater proportion of colonies added spots to their hive fronts after exposure to VG extracts than was observed for colonies that received the sham control (Fig 5; Fisher’s exact test: p = 0.001). A small number of colonies had dislodged their squares of filter paper by the end of the 6-hour test (2 of 19 VG-extract colonies and 4 of 20 control colonies). Of the colonies with papers remaining, a greater proportion of papers that were soaked in VG extract had spots on them than papers soaked in ether only (Fig 5, Fisher’s exact test: p = 0.02). However, we note that colonies exposed to VG extracts were more likely to respond by placing spots on hive fronts rather than on test papers (Fig 5, Fisher’s exact test: p = 0.03).

### Fecal spotting repels *Vespa soror* from nest entrances

**Fig 6:**
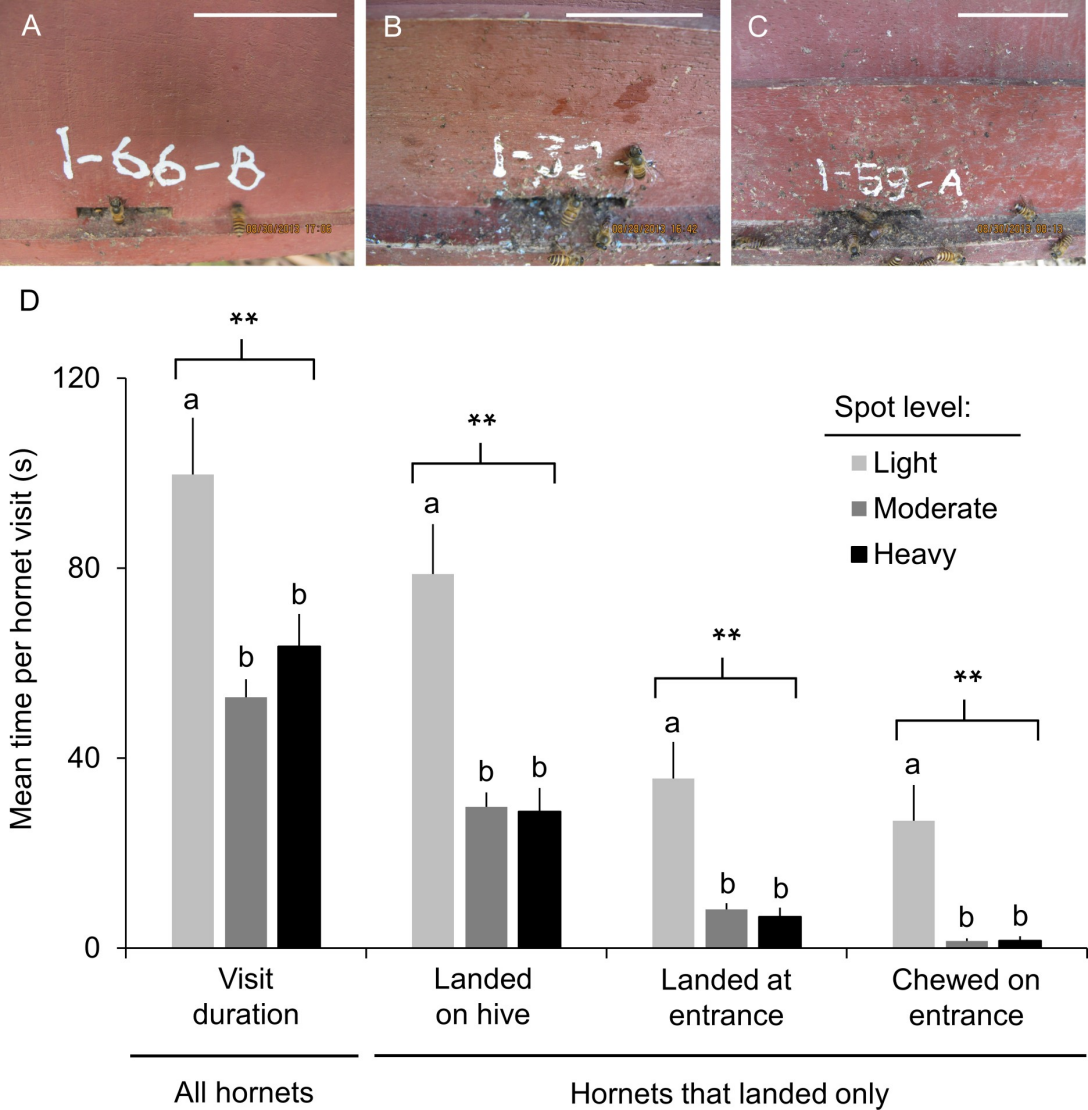
*Vespa soror* workers spent less time trying to breach nest entrances as spotting increased. Images show the spread of spots around entrances for colonies that ranked at the midpoint of each of the three spotting categories: (A) light spotting (median 42, range 8–99 spots per hive front); (B) moderate spotting (median 275, range 107–495 spots per hive front); (C) heavy spotting (median 636, range 504–1,523 spots per hive front). White scale bars = 5 cm. (D) Mean time (± SEM) per hornet spent visiting a colony and, for hornets that landed on hives, time spent performing nest-breach behaviors (i.e., landed anywhere on the hive, landed at the entrance specifically, chewed on the entrance margins). Visit duration was calculated for all hornets (left: light = 81 visits; moderate = 141 visits; heavy = 54 visits). Time spent trying to breach nests was calculated only for hornets that landed on hives (right: light = 72 visits; moderate = 115 visits; heavy = 46 visits). Data were obtained from the videos used to produce Table 2 (see legend). Asterisks indicate a significant difference among groups identified within brackets (** p < 0.0001); letters indicate differences among treatment means).

The severity of attacks by *V*. *soror* decreased in multiple ways as level of spotting around nest entrances increased (Fig 6A–6C shows colonies with median levels of spotting in lightly, moderately, and heavily spotted categories; see Supporting Information for details about categories). Overall, the average duration of a hornet’s visit was significantly shorter for colonies with moderate or heavy levels of spotting compared to colonies with light spotting (Fig 6D; ANOVA: F_2, 273_ = 11.7, p < 0.0001). Although visiting hornets were equally likely to land anywhere on hives regardless of degree of spotting (Table 2), those that landed spent less time on hives that were moderately or heavily spotted (Fig 6D; ANOVA: F_2, 230_ = 16.1, p < 0.0001). Furthermore, there was a strong effect of spotting on the frequency and duration of behaviors that would result in hornets breaching nests. Firstly, hornets were increasingly less inclined to land at entrances or chew on them as colonies became more heavily spotted (Table 2). Secondly, if a hornet did land on a hive, she spent less time landed at or chewing on hive entrances when they had moderate or heavy spotting (Fig 6D; land, ANOVA: F_2, 230_ = 13.9, p < 0.0001; chew, ANOVA: F_2, 230_ = 12.4, p < 0.0001). With increased spotting, the reduction in time spent by hornets focused on entrances was substantial. On average, the duration of time landed at an entrance per visiting hornet was reduced by 77–81% and the time spent chewing was reduced by 94% if the hive fronts of colonies were moderately or heavily spotted rather than lightly spotted. Lastly, lightly spotted colonies were most likely to experience attacks that involved multiple hornets (Table 2). While behaviors related to nest breach became less frequent with increased level of spotting, it did not change the percentage of visits during which a hornet killed a bee (Table 2).

**Table 2:** Moderate or heaving spotting reduced multiple-hornet attacks and nest breach behaviors by *Vespa soror* workers. Data were obtained from videos of hive visits by *V*. *soror* workers that were recorded in the same apiary over a three-day period (the source of data for Fig 6). For landing, chewing, or killing, data were collected from videos for individual hornets to determine the percentage of all hornets that performed these attack behaviors while visiting colonies that were lightly (n = 81 hornets), moderately (n = 141 hornets), or heavily spotted (n = 54 hornets). For incidence of multiple-hornet attacks, the percentage of videos that recorded attacks on lightly (n = 61 videos), moderately (134 = videos), or heavily spotted (n = 47 videos) colonies that included more than one hornet was determined (here, each video was a replicate). Because a single video could yield data for several hornets if it recorded a multiple-hornet attack, the total number of attack videos was lower than the total number of hornet visits. Asterisks indicate behaviors that are required to breach hive entrances and occupy nests.

Behavior during attack	% attacks with behavior observed			Chi-square test of independence
	Light spotting	Moderate spotting	Heavy spotting	
Landed anywhere on hive	88.9	81.6	85.2	χ^2^ = 2.1, df = 2, p = 0.34
* Landed at entrance	69.1	53.9	44.4	χ^2^ = 8.8, df = 2, p = 0.01
* Chewed on entrance margins	28.4	8.5	7.4	χ^2^ = 19.3, df = 2, p < 0.0001
Killed bee during interaction	28.4	31.9	31.5	χ^2^ = 0.3, df = 2, p = 0.85
* Multiple-hornet attack	24.6	6.7	14.9	χ^2^ = 12.2, df = 2, p = 0.002

## Discussion

Our study documents a remarkable defense employed by *Apis cerana* honey bees in response to attack by the group-hunting giant hornet, *Vespa soror*. *A*. *cerana* workers in Vietnam collect filth (primarily animal feces in this study) and apply it in mounded spots around hive entrances in response to natural attacks by this formidable hornet predator. This response was sustained for several days after hornet attacks ceased and, for some colonies, resulted in a substantial coating of filth that extended outward from nest entrances. Exposure to the hornets’ VG extracts only (putative nest-marking pheromones [20]) also initiated a spotting response, although not as strongly as natural hornet attacks. A colony with moderate or heavy spotting on its hive (Fig 6B and 6C) had a reduced likelihood of visiting hornets landing at and chewing on its entrance, which was the only accessible entry point for attackers. If hornets did land on a hive, moderate to heavy spotting significantly reduced the time hornets spent attempting to breach the nest by landing near the entrance or chewing on it. Increased spotting also resulted in a lower percentage of attacks that involved multiple hornets. In contrast, *A*. *cerana* workers did not apply feces around nest entrances when they were attacked by *Vespa velutina*, which we show rarely landed on hives, never chewed on entrance margins, and killed fewer bees during attacks in comparison to *V*. *soror*. The difference in the defensive response by bees to *V*. *soror* and *V*. *velutina* likely reflects the hornets’ respective hunting tactics and the level of threat they pose to colonies because of the intensity of their attacks. *V*. *velutina* is well known for hunting individually and hawking single bees outside of nest entrances, typically while both predator and prey are in flight [9], but *A*. *cerana* defensive behaviors effectively limit their damage as predators [9, 16]. In contrast, while we observed *V*. *soror* workers flying away with single bees, they frequently recruited conspecifics and executed mass attacks that were focused on gaining entry into *A*. *cerana* nests, with the potential for imminent and large-scale destruction of colonies if nests were breached. We allowed one attack to proceed without disruption and confirmed that successful nest entry by *V*. *soror* was followed by rapid occupation of the nest and predation of bee brood (the surviving bees absconded), which is the well-known outcome of the damaging and often lethal attacks on honey bees reported for the other giant hornet, *Vespa mandarinia* [7, 9, 20]. This finding extends previous observations of observed mass attacks by *V*. *soror* on nests of social wasp species [50].

The collection of solid filth (predominantly feces in this study) is a previously unknown behavior for *Apis*. It is widely recognized that the only solid materials honey bees collect from plants are pollen and resin, which they transport home in their corbiculae on their hind legs [61]. Several species of honey bee have been observed collecting urine and liquids from animal feces, but they drink fluids at these resources, presumably to obtain nutrients [52, 54, 55]. Among the social bees, only stingless bees are known to collect filth solids, including mammal feces for use in nest construction [56, 59, 60]. We report here for the first time that *A*. *cerana* workers forage for and carry away in their mandibles solid clumps of animal feces (bird and mammal), which they apply around nest entrances when they return home. We have no evidence that workers alter this material once it is foraged or manipulate it in any way other than shaping clumps into spots that they apply around entrances. There would be little time to do so because marked foragers applied mounded spots to hive fronts shortly (<2 minutes) after leaving dung piles at the apiary’s edge.

Spotting behavior by *A*. *cerana* is widespread throughout Vietnam and has been reported to us in southeastern China (Hunan and Yunnan Provinces), Thailand, Bhutan, and Nepal (Z. Huang, K. Klett, R. Gregory, and S. Joshi; pers. communication). Spots in photographs from these additional locations appeared similar to the spots we observed in Vietnam that were composed of feces and other filth (and potentially other unknown materials). These locations fall within the range of *V*. *mandarinia*, the sister species of *V*. *soror* [35, 41, 42, 49], making it likely that spotting occurs in response to mass attacks by *V*. *mandarinia* as well. Fecal spotting is behaviorally analogous to observations of “plant smearing” by *A*. *cerana japonica* in Japan, which occurs in response to attack by *V*. *mandarinia* [45]. In this recently described behavior, workers carry gnawed plant material in their mandibles and then smear their juices around nest entrances, leaving dark stains. Although we did not study how filth foraging is organized, we observed several workers performing “emergency” dances outside of hive entrances, a behavior that recruits nestmates to smear plant material in Japan [46]. It is fascinating that *A*. *cerana* has been observed foraging for plant material in the northern part of its range and for filth (feces) in the southern part of its range to defend nests against attack by different, but equally deadly, mass-attacking *Vespa* predators. It suggests that *A*. *cerana* has evolved a unique strategy for collective defense against giant hornets that is qualitatively different from balling [18, 20, 34] or body shaking [22, 27], two behaviors it shares with other *Apis* species [4], but that there may be flexibility regarding the features of this novel defense. This flexibility could come from regional differences in choice of material or undocumented diversity across *A*. *cerana*’s range in the materials that workers collect to defend their colonies.

By what mechanism does fecal spotting protect *A*. *cerana* colonies from mass attack by *V*. *soror*? There are several ways this defense could work. Our study shows that when colonies were attacked, spots suppressed the tendency of attackers to interact with nest entrances. In comparison to a lightly spotted colony, hornets targeting a heavily spotted colony often appeared reluctant to contact especially spotty areas around entrances, even though their movements suggested they knew where entrances were located (S5 and S6 Videos). It is possible that fecal spots contain compounds that are repellent to *V*. *soror* attackers, which must approach and then chew their way into the small entrances of wooden hives or tree cavities, and pass through the tunnel-like entrances that characterize *A*. *cerana* nests in the wild [5]. Repellent properties could be inherent to feces itself, such as cues that are associated with vertebrate predators [62] or cues that initiate feces avoidance to limit parasite or disease transmission [63, 64]. Earwigs, for example, release volatiles that smell like feces to limit predation [65] and *Manduca sexta* caterpillars defecate profusely on themselves as a defense against attack [66]. Alternatively, repellency may stem from properties that repel insects via numerous potential modes of action [67, 68], but are not feces specific or found in all fecal resources. Larvae of many chrysomelid beetle species repel insect and bird predators using fecal shields that they wear on their backs, in which plant defensive compounds that larvae have consumed are recycled [69–75]. It is possible that filth foragers in our study sought specific compounds in the feces they collected because marked individuals often returned to the same sites on dung piles and pulled at material for a while before carrying away clumps. These compounds could be present in feces as a result of the dung producers’ bodily processes or because they were in the food that they ate (e.g., plant material) and remained present in their feces. The plants workers smeared on hives in response to *V*. *mandarinia* attack in Japan belong to groups that produce compounds that are known to repel insects [46, e.g., 76, 77] and are also used in chrysomelid fecal shields [75]. It is also possible that *A*. *cerana* forages for plant-derived defensive compounds both from plants directly [45, 46] and from the feces of herbivores that eat them (our study) in response to attack by giant hornets. While further study is needed to determine the proximate mechanisms by which fecal spotting repels entrance-focused attacks by *V*. *soror* workers, what is just as interesting is that *A*. *cerana* workers forage on feces and appear immune to these aversions.

Fecal spotting helps to slow or limit the breach of nests by attacking wasps, but it is also possible that the application of filth around nest entrances could reduce the likelihood of attacks transitioning from single-hornet visits to multiple-hornet affronts. Although we saw multiple-hornet attacks on moderately and heavily spotted colonies, the percentage of visits that were by multiple hornets was highest for colonies with the least spotting. By what mechanisms could fecal spots suppress hornet recruitment? We showed that applying extracts from hornets’ glands initiated spotting, so one possibility is that fecal spots mask the marking pheromones of hornet scouts. While not confirmed, it is currently presumed that *V*. *mandarinia* workers follow a chemical trail created by scout nestmates to locate target colonies [20]. We have observed *V*. *soror* workers vigorously rubbing their glands on hives and on vegetation above them, as have others [50], suggesting that they too follow odor trails to target colonies. If fecal spots mask these marking pheromones, then it would make it more difficult for scouts to recruit nestmates to colonies for mass attack. If pheromone masking works, our evidence suggests that *A*. *cerana* may accomplish this by spotting around entrances generally rather than spotting directly on sites where pheromones were deposited by scouts because spots were more often applied to hive fronts than to filter papers soaked in VG extracts and pinned at hive entrances. Furthermore, we failed to document spotting on sites where hornets rubbed their glands when they were far away from nest entrances (e.g., the top or sides of hives). Additionally, our finding that spotting persisted for days after attacks ceased suggests that its function extends beyond simply masking scout pheromones. Finally, it is also possible that fecal spots mask the odor of colonies, which might disrupt recruitment to targets because colony odor is used by hornets, in addition to visual cues, to locate their prey [78–80]. Much remains to be learned about the potential for fecal spotting to prevent the onset of a mass attack by suppressing hornet recruitment, in addition to repelling attacking hornets from entrances.

Is the use of feces by *A*. *cerana* an example of tool use? Tool use is considered widespread among insects [81–83] and feces is routinely cited as a tool that is used by insects and a variety of other animal taxa [81, 83, 84]. The definition of tool use has been controversial historically and efforts at refinement often accept, reject, or clarify elements of previous definitions [81–83, 85–89]. A recent and detailed definition comes from Shumaker and colleagues [83], who updated Beck’s [87] influential description of tool use to include four key criteria. We believe fecal spotting meets their definition of tool use, as we will explain through careful paraphrasing of their requirements. First, the user must externally employ an environmental object, which in this case is a piece of feces or other filth. Secondly, the user must alter (with “purposiveness” and “more efficiently”) the form or condition of another object; we argue that fecal spots enhance the properties of the nest surface as a defensive barrier by reducing the likelihood of hornets engaging in behaviors that lead to nest breach by multiple attackers. Third, the user must hold or directly manipulate the tool before or during use, which a forager does when she carries feces from a dung pile and shapes it with her mandibles into spots on the surface of her nest. Finally, the user must effectively orient the tool, which a forager does when placing fecal spots around her nest entrance, the focus for nest breach by attacking hornets. Here, “apply” is the relevant mode of tool use (i.e., attach to a surface without adhesive) and “detach” then “reshape” is the mode of tool manufacture (i.e., remove it from the dung pile, which from the bee’s perspective has a fixed connection to the substrate, then restructure it) [83]. A similar analysis supports the manufacture of leaf baffles by tree crickets as an example of tool use [90]. Fecal spotting is analogous to the use of fecal shields by many chrysomelid beetles to deter predators or the application of mud to antlers by deer stags to attract mates, both of which are categorized with confidence as tool use [83]. Furthermore, another influential definition emphasizes that one goal of tool use is to mediate the flow of information between the tool user and other organisms [88], which *A*. *cerana* does if fecal spots mask hornets’ recruitment chemicals or repel attacking hornets. Finally, fecal spotting conforms to the most recent attempt to seek a universal definition of tool use, which simply states that tool use is the deliberate use of an object outside of one’s body to perform an intermediate task that advances the user’s goal [89]. Thus, collection of feces and other filth materials from the environment and their application to nest surfaces for the purpose of defense by *A*. *cerana* meets current conceptions of tool use.

In summary, we have documented fecal spotting as a novel defensive behavior that is used by *Apis cerana* honey bees to defend their nests against attack by the giant hornet *Vespa soror*, a poorly studied but formidable vespid that employs a mass-attack strategy similar to that used by its better-known sister species, *V*. *mandarinia* [7]. Viewed within the spectrum of counterstrategies that honey bees have evolved to defend their nests against a diverse array of threats [4], fecal spotting stands out as extraordinary for several reasons. It marks the first report of honey bees of any species foraging for materials that are not derived from plants or water-based fluids (excluding *A*. *florea* colonies salvaging their own beeswax [51]). It is also the first clear-cut example of honey bees using a tool in nature (nests and the materials used to construct them are not generally considered tools [82, 83], although some authors disagree [81, 89, 91]). Curious defensive behaviors within *Apis* tend to be innovatively threat specific [3]. In that context, we show here that fecal spotting by *A*. *cerana* is directed at *V*. *soror* hornets that attack in groups with the ruinous objective of occupying nests to prey on bee brood, but not at *V*. *velutina* hornets that prey on individual adult bees outside of nests only. However, much remains to be understood about this predator-prey interaction. What evolutionary steps underpinned the switch to foraging for filth (a new forage category) in the context of collecting tools for predator defense (a new function for foraging)? What properties of feces provide *A*. *cerana* colonies with a measure of protection against *V*. *soror* attacks? How is spotting behavior organized within *A*. *cerana* workforces? Despite exerting enormous selective pressure on honey bees as one of their most deadly predators, remarkably little is understood about how *V*. *soror* or *V*. *mandarinia* workers recruit nestmates or coordinate their attacks. Thus, basic aspects of the behavioral ecology of both predator and prey remain to be discovered before the role that feces plays in thwarting mass attacks by giant hornets can be fully appreciated. These questions are more pressing with the recent introduction of giant Asian hornets to western North America [92–95]. The often negative consequences of the establishment of honey bee or hornet species into regions where predator-prey arms races have not had sufficient time to co-evolve highlight the importance for honey bees of having nest defenses that are tailored to meet the threats that different hornet species pose to their survival [8, 9, 26, 40, 44].

## Materials and methods

We conducted all field work in three apiaries that were near each other (<1.4 km apart) within the Ba Trai Commune, Ba Vì District, Hanoi Province, Vietnam (GPS coordinates, Apiary 1: 21.118, 105.335; Apiary 2: 21.106, 105.336; Apiary 3: 21.105, 105.335; see S1 Fig). Colonies were managed by local beekeepers and housed in pairs in wooden hives; the two colonies sharing a hive were physically separated and used different entrances on opposite ends of the box (thus, they did not share the same hive front; n = 136, 148, and 55 colonies in Apiaries 1–3, respectively). All the colonies had three frames and hives were similar in size; colony and hive setups were typical of managed *A*. *cerana* colonies in this region of Vietnam. While entrance size was not standardized, they were all small (3–6 cm wide and <1 cm high) to hinder hornets from entering hives. Hive-front walls, entrances, and landing boards were measured so that distance and area could be determined in photographs and videos. All field work was conducted from August 14 to October 10, 2013, when predation of *A*. *cerana* bees by *V*. *soror* hornets and other *Vespa* species was expected (according to local beekeepers). Daytime temperatures were consistently warm over that time (20–35 ^o^C) and favorable for foraging by both bees and hornets.

### Ethics statement

The field studies described in this paper involved observation of free-living animals. No protected or endangered species were sampled and we did not harm bees or hornets while observing their natural interactions. We received permission to conduct our research from the beekeepers on whose property the study apiaries were located, as well as the People's Committee of Tây Đằng Town, Ba Vì District. Because the beekeepers had land use rights on these properties according to Vietnamese law, no permits were required once permissions were granted. Gland extracts were obtained from hornets purchased from a commercial wasp farmer; hornets were not collected from the wild by anyone on the research team.

### Do *Apis cerana* foragers collect feces and apply it as spots around nest entrances?

We first determined that the spots we had observed on hive fronts were placed there by bees and that they were added over time. At the start of the study, we cleaned all hive fronts (with a scrub brush and water until spot free) in all apiaries on the same day (day 0, August 14), then we returned to document over a 10-day observation period the absence or presence of spots on hives (on days 5 and 10). Photographs were taken only of hive fronts because spots were not observed elsewhere. The spread of the spots on each hive was estimated by determining the position of the farthest spots on the vertical (top and bottom) and horizontal (right and left) margins of each hive front. A rectangle was drawn using these four locations as its boundary to estimate the area of spot spread, the distance of the farthest spot to the closest margin of the entrance opening, and whether the spread of spots encompassed the hive entrance. Photographs were analyzed using ImageJ (U.S. National Institutes of Health, Maryland) and distances were measured by reference to known widths of entrances. The hives were painted dark red, green, or light blue, so spots (generally shades of brown and gray) were visually identifiable on the front of hives. On day 7, we videorecorded two hive fronts in Apiary 2 from 09:00–17:00 to confirm that the spots that appeared throughout the day were applied by bees (digital HD video camera, Sony Handycam HDR-PJ340).

This work made clear that colonies in Apiary 1 had the highest level of spotting among those in the three apiaries, so most of our subsequent studies were conducted there. We suspected that the spots that bees applied to hive fronts were animal feces (based on their smell and beekeepers’ observations), so we next determined whether *A*. *cerana* workers would forage on piles of fresh dung (chicken, pig, water buffalo, and cow) that were placed at the edge of the apiary. Over a two-day period (August 24 and 25), observers marked bees with paint as they landed on dung piles, while other observers monitored hive fronts for marked bees; their behavior was noted at both locations.

Finally, in late August 2013 (when *V*. *soror* workers frequently visit apiaries), we surveyed beekeepers throughout Vietnam who were known to an extension specialist from the Vietnam Bee Research and Development Centre in Hanoi. We contacted them by phone and asked each beekeeper what species of honey bee they kept, the number of colonies in their apiaries, the current presence of spots on hives, and the estimated incidence of absconding due to hornet attack over the last two years. From these data, we generated a map showing known occurrences of spotting throughout the country.

### Does *Apis cerana* respond with fecal spotting to attacks by *Vespa soror*?

#### Test 1: Fecal spotting during attack

To determine whether visitation by *V*. *soror* hornets prompted fecal spotting by workers in *A*. *cerana* colonies, we compared differences in spotting between colonies in Apiary 1 that were either attacked by *V*. *soror* over a day or left unmolested. During a two-hour period (07:00–09:00) in the early morning of August 22, on day 8 of the aforementioned 10-day observation period, we identified colonies that were being visited by *V*. *soror* hornets (n = 12 colonies). Over the same period, another group of control colonies was identified that were not visited by hornets (n = 14 colonies). Attacks on the first set of colonies were permitted to continue naturally for the remainder of the day and six observers recorded minute-by-minute arrival and departure times of hornets to determine the cumulative number and duration of hornet visits per colony. Two other observers monitored control colonies to ensure they were not visited by hornets. Colonies were in close proximity and hornet visits were separated in time, thereby permitting continuous monitoring of the 26 focal colonies. If hornets entered the vicinity of control colonies, they were prevented from approaching hives by waving plastic bags tied to 2 m sticks at them, which caused them to fly away quickly (observers stood away from hive entrances and colonies appeared undisturbed by hornet-human interactions). Hive fronts of all colonies were photographed at 2-hour intervals over the first day (between 09:00 and 17:00) and for a final time the next morning (07:00). The number of spots on hive fronts was estimated by counting spots within the squares of a 1x1 cm^2^ grid that was superimposed over photographs of each hive front, scaled according to known lengths of entrances (ImageJ; assumed 10 spots/cm^2^ if filled). Change in mean spot number was compared over time and between treatments.

#### Test 2: Fecal spotting after attack

We conducted a second test over three days to determine whether the application of spots persisted even after *V*. *soror* attacks had ceased. On August 24 (the end of the aforementioned 10-day observation period), we again cleaned hive fronts. On August 25, we identified attacked and no-attack control colonies by seeing which colonies were visited by *V*. *soror* hornets early in the morning, as described for Test 1 (n = 22 attack and 6 control colonies; we excluded colonies used for Test 1). We allowed hornets to continue to visit “attacked” colonies (and prevented them from visiting control colonies), but for the second and third days thereafter (August 26 and 27), hornets were waved away from *all* colonies to determine the persistence of an attacked colony’s spotting response in the absence of further attack. As in Test 1, six observers recorded minute-by-minute arrival and departure times for hornets as they visited “attacked” colonies throughout the first day of the test. Hive fronts were photographed at the end of each of the three days and spot counts on hive fronts were estimated (as in Test 1) to compare change in mean spot number over time and between the two treatments.

#### Test 3: Fecal spotting in response to hornet species

We compared differences in spotting response to attack by *V*. *soror* versus *V*. *velutina* to determine whether spotting was in response to attack by *V*. *soror* specifically. On the morning of September 16, we identified 22 colonies in Apiary 1 (where *V*. *soror* was often observed) and 19 colonies in Apiary 3 (1.4 km away, where *V*. *soror* was rarely observed) that were attacked by *V*. *soror* and *V*. *velutina*, respectively. Once these colonies were identified, we allowed the attacks to continue for two days (September 16 and 17). These colonies were protected from attack by hornets of the other species by waving them away over these two days (as in Tests 1 and 2). We cleaned hives before the test started and we also waved away all hornets for two days in advance of the test to minimize residual spotting due to potential recent exposure to hornets. The mean number of spots on hive fronts was compared between the two groups based on photographs of hive fronts taken at the end of the second day (spot counts were estimated as in Test 1).

As a complement to this third test, we determined differences in the attack behaviors of *V*. *soror* and *V*. *velutina* workers. Attacks were observed over six days per species (three days concurrently) as hornets visited colonies in Apiary 1 between August 23 and September 8. During each day of observation, hornet visits were monitored from 07:00 to 17:00 by six observers stationed throughout the apiary, who monitored attacks as they encountered them. An attack was characterized if a hornet visited a hive for at least 30 seconds. During such a visit, we recorded whether the focal hornet performed any of the following behaviors: hovering in front of the hive, landing anywhere on the hive or at the entrance specifically, chewing on the margins of entrance opening, self-grooming, fanning wings, performing trophallaxis with a conspecific, rubbing abdomen on the hive, and chasing or killing bees. Visit duration was estimated in 30-second increments. In total, we characterized 857 visits by *V*. *soror* workers and 328 visits by *V*. *velutina* workers.

#### Test 4: Fecal spotting in response to gland extracts

Throughout our field studies, we frequently observed *V*. *soror* workers rubbing their abdomens on hives and nearby vegetation during attacks. This behavior is similar to how *V*. *mandarinia* scouts putatively mark nests for mass attack using pheromones from their van der Vecht gland (VG) [20], which is located on the last abdominal sternite of workers in many *Vespa* species [96, 97]. We wondered whether secretions deposited during abdomen rubbing by *V*. *soror* workers induced spotting by *A*. *cerana* colonies. To examine this idea, we presented colonies in Apiary 3 –where spotting was least frequent–either with extracts from van der Vecht glands (VG) or with an ether sham (control). On September 15, we ranked colonies by strength (assessed by totaling the estimated % coverage of each comb surface by bees [98]) and then, within successively ranked pairs, we randomly divided colonies into two treatment groups. We cleaned all hive fronts at that end of the day and, the next morning, we presented colonies with a 1x1 cm^2^ piece of filter paper that had been soaked either in ether containing VG extract or in ether alone (n = 20 and 19 colonies respectively). Prior to starting this test, we obtained *V*. *soror* workers from a vespiary in Ngoc Dong, Yen Lap District, Phu Tho Province, Vietnam (GPS coordinates: 21.242, 105.150). Each VG extract was created by dissecting the last abdominal sternites from three chilled hornet workers, placing these sternites for 24 h in a vial that contained 0.5 mL ether, then repeatedly soaking and drying a square of filter paper in the vial until there was no ether left (following [20]; each colony was assigned its own vial containing VGs from three hornets). Control colonies receiving the ether-only sham treatment were presented with filter papers that had been repeatedly soaked and dried in ether only. After the ether had vaporized from a square of filter paper, it was pinned to the landing board in the middle of a colony’s hive entrance. Hive fronts were photographed at the start and end of the six-hour test and spot number was determined from the photos, as described previously. Mean spot number was compared between treatments; the presence of spots on each filter paper was also noted.

### Does fecal spotting affect *Vespa soror* behavior?

We monitored visits by *V*. *soror* hornets to *A*. *cerana* colonies to determine whether hornet behavior was affected by the level of fecal spotting on hives. For three days (August 28–30), eight observers stationed throughout Apiary 1 videorecorded hornets as they visited colonies. Care was taken to start recordings as close as possible to the initial approach of hornets to hives and from a sufficient distance (>2 m) that observers did not disturb hornet-bee interactions. For each focal hornet, we determined total visit duration, time spent landed anywhere on the hive or at the entrance specifically (i.e., when any part of the hornet’s body overlapped with the entrance opening), time chewing on the margins of the entrance opening, and whether or not the hornet killed a bee. If a single video contained multiple hornets, then each hornet’s behavior was assessed separately. The number of hornets in each recording was also noted to assess the occurrence of multiple-hornet attacks.

Hive fronts were cleaned four days before the start of these behavioral observations and spots were permitted to accumulate naturally thereafter. Because spot number changed over the three days that hornet visits were videorecorded, we photographed hive fronts at the start and the end of each day and we estimated spot number on the front of a hive during a hornet’s visit using the image captured closest to each hornet’s arrival. Spots were counted using a scaled grid superimposed on photographs in ImageJ, as previously described. Each hornet visit was treated as an independent experimental replicate because spot number changed over time and visiting hornets were not identifiable. Attacked colonies were subsequently categorized as being spot free (n = 4 hornet visits), or having light (1–100 spots; n = 81 hornet visits), moderate (101–500 spots; n = 141 hornet visits), or heavy spotting (500+ spots; n = 54 hornet visits). These four categories captured the range of fecal spotting we observed on hive fronts in our study apiaries. Category boundaries were established prior to data collection, which is why sample sizes were not similar across categories. We prioritized broad categories, with only two categories between heavy spotting and no spotting, so that bins had biological relevance (i.e., reflected differences in strength of response), but were not too finely parsed. Because spotting was pervasive in Apiary 1 by the end of August, the spot-free category was excluded from the analysis due to small sample size, as were 63 visits in the other categories because of poor video quality (e.g., inadequate perspective or the recording was too brief to assess hornet behavior; not included in sample sizes). A total of 276 hornet visits recorded in 242 attack videos were used in the analysis (86 different colonies were visited during these attacks).

### Statistics

ANOVAs and t-tests were performed using SAS (v. 9.3, SAS Institute). One-way ANOVAs (proc GLM) were used to compare hornet behavior among spotting categories and repeated-measures ANOVAs (proc mixed) examined changes in spot number over time. If models were significant, then means were separated by the Tukey HSD test using the Tukey-Kramer method. Differences in spot number in response to *V*. *soror* versus *V*. *velutina* and VG extract versus control were compared using two-tailed t-tests. Contingency tables (2x2 and 2x3) were used to explore frequency distributions. The presence of spots was compared between VG-extract and control treatments using Fisher’s exact test (because some cell sizes were <5; www.vassarstats.net). Otherwise, chi-square tests of independence were used to compare percent spotting across the three study apiaries and hornet behavior across the three categories of spotting. Two-tailed Z-tests compared the equality of the proportion of *V*. *soror* versus *V*. *velutina* exhibiting different types of attack behaviors (www.socialscistatistics.com). The significance level of all tests was set at α = 0.05.

## Supporting information

S1 Fig Map of Vietnam showing locations were fecal spotting was reported on hives. The map indicates locations where beekeepers who kept *Apis cerana* colonies reported either the presence (▲) or absence (✕) of spots on the front of their hives. The location of our study apiaries is also shown (★). The inset map (upper right) shows the part of central Vietnam that is enlarged in the main map. The map was generated using free vector and raster map data from Natural Earth (public domain maps; naturalearth.com).
(TIF)

S1 Video An *Apis cerana* worker foraging on chicken dung carries it away using her mandibles. (M4V)

S2 Video An *Apis cerana* worker foraging on chicken dung. (M4V)

S3 Video An *Apis cerana* worker applying and shaping a fecal spot near a hive entrance. (M4V)

S4 Video An *Apis cerana* worker applying a fecal spot near a hive entrance after being paint marked on a dung pile near the apiary. (M4V)

S5 Video A multiple-hornet attack by *Vespa soror* on a lightly spotted *A*. *cerana* colony. (M4V)

S6 Video A multiple-hornet attack by *Vespa soror* on a heavily spotted *A*. *cerana* colony. (M4V)

S1 Data Excel file of data used for all figures and tables. (XLSX)
