# Supplementary figures and images for: Honey bees (Apis cerana) use animal feces as a tool to defend colonies against group attack by giant hornets (Vespa soror)
Source: PLoS One. 2020 Dec 9;15(12):e0242668. doi: 10.1371/journal.pone.0242668 (PMC7725375; doi:10.1371/journal.pone.0242668)

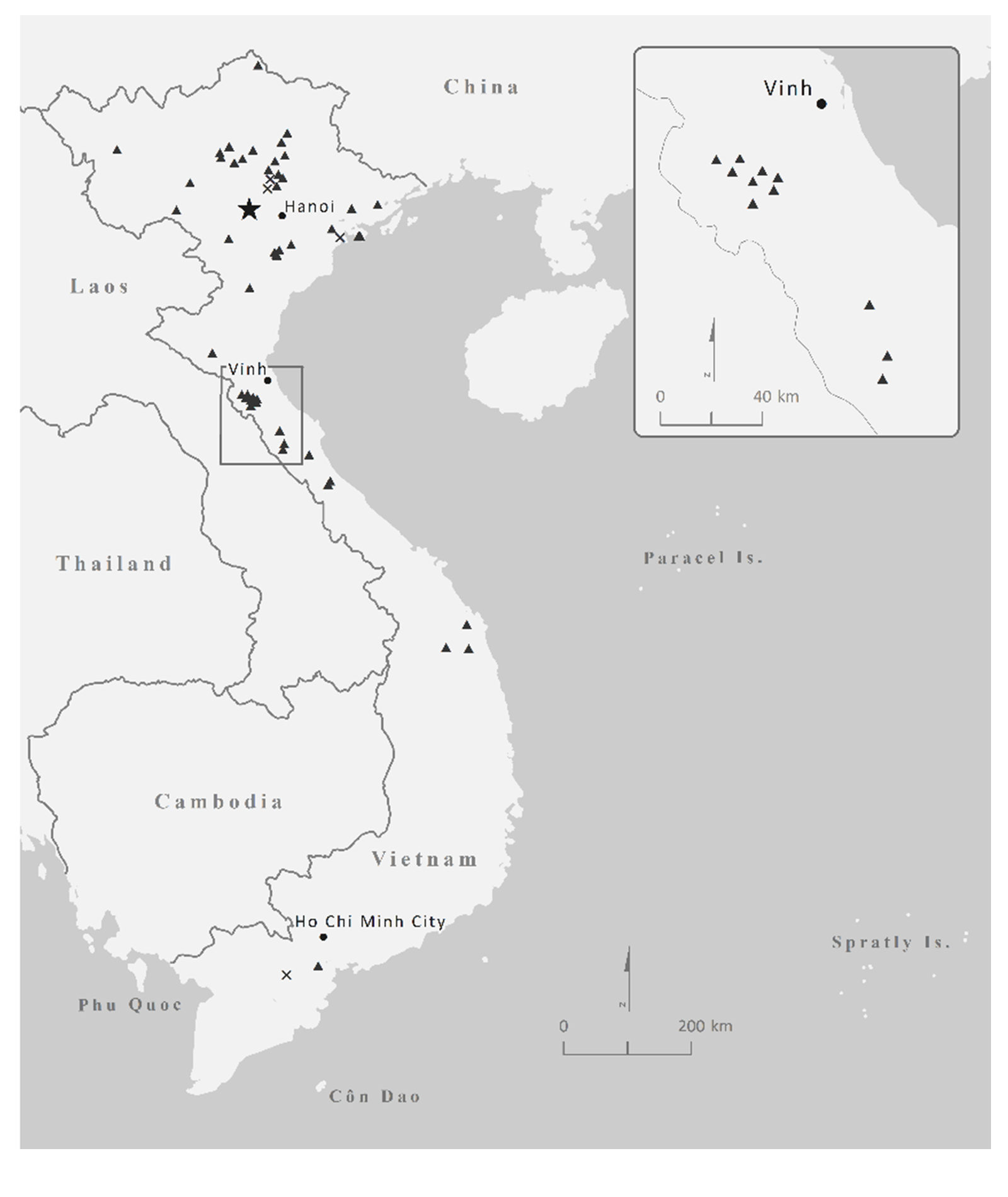

Supplement: S1 Fig — The map indicates locations where beekeepers who kept Apis cerana colonies reported either the presence (▲) or absence (✕) of spots on the front of their hives. The location of our study apiaries is also shown (★). The inset map (upper right) shows the part of central Vietnam that is enlarged in the main map. The map was generated using free vector and raster map data from Natural Earth (public domain maps; naturalearth.com). (TIF) [file pone.0242668.s001.tif]
